# Economic evaluation of meningococcal vaccines: considerations for the future

**DOI:** 10.1007/s10198-019-01129-z

**Published:** 2019-11-21

**Authors:** Hannah Christensen, Hareth Al-Janabi, Pierre Levy, Maarten J. Postma, David E. Bloom, Paolo Landa, Oliver Damm, David M. Salisbury, Javier Diez-Domingo, Adrian K. Towse, Paula K. Lorgelly, Koonal K. Shah, Karla Hernandez-Villafuerte, Vinny Smith, Linda Glennie, Claire Wright, Laura York, Raymond Farkouh

**Affiliations:** 1 Population Health Sciences, Bristol Medical School, University of Bristol, Bristol, BS8 2BN UK; 2 Health Economics Unit, University of Birmingham, Birmingham, B15 2TT UK; 3 Université Paris-Dauphine, PSL Research University, LEDa [LEGOS], 75775 Paris, France; 4 Department of Pharmacy, University Medical Center/University of Groningen, 9712 CP Groningen, The Netherlands; 5 Department of Health Sciences, University Medical Center/University of Groningen, 9712 CP Groningen, The Netherlands; 6 Department of Economics, Econometrics and Finance, University Medical Center/University of Groningen, 9712 CP Groningen, The Netherlands; 7 Department of Global Health and Population, Harvard T. H. Chan School of Public Health, Harvard University, Cambridge, MA 02115 USA; 8 Institute of Health Research, Medical School, University of Exeter, Exeter, EX1 2LU UK; 9 School of Public Health, Bielefeld University, 33615 Bielefeld, Germany; 10 Centre on Global Health Security, Royal Institute of International Affairs, London, SW1Y 4LE UK; 11 FISABIO-Public Health, 46020 Valencia, Spain; 12 Office of Health Economics, London, SW1E 6QT UK; 13 Meningitis Research Foundation, Newminster House, 27-29 Baldwin Street, Bristol, BS1 1LT UK; 14 Vaccine Medical Development, Scientific and Clinical Affairs, Pfizer Inc, Collegeville, PA 19426 USA; 15 Pfizer Inc, Collegeville, PA 19426 USA; 16 German Cancer Research Center (DKFZ), Heidelberg, Germany

**Keywords:** Meningitis, Meningococcal, Vaccine, Cost-effectiveness, QALY, I. Health, Education, and Welfare, I.18 Government Policy, Regulation, and Public Health

## Abstract

In 2018, a panel of health economics and meningococcal disease experts convened to review methodologies, frameworks, and decision-making processes for economic evaluations of vaccines, with a focus on evaluation of vaccines targeting invasive meningococcal disease (IMD). The panel discussed vaccine evaluation methods across countries; IMD prevention benefits that are well quantified using current methods, not well quantified, or missing in current cost-effectiveness methodologies; and development of recommendations for future evaluation methods. Consensus was reached on a number of points and further consideration was deemed necessary for some topics. Experts agreed that the unpredictability of IMD complicates an accurate evaluation of meningococcal vaccine benefits and that vaccine cost-effectiveness evaluations should encompass indirect benefits, both for meningococcal vaccines and vaccines in general. In addition, the panel agreed that transparency in the vaccine decision-making process is beneficial and should be implemented when possible. Further discussion is required to ascertain: how enhancing consistency of frameworks for evaluating outcomes of vaccine introduction can be improved; reviews of existing tools used to capture quality of life; how indirect costs are considered within models; and whether and how the weighting of quality-adjusted life-years (QALY), application of QALY adjustment factors, or use of altered cost-effectiveness thresholds should be used in the economic evaluation of vaccines.

## Introduction

Invasive meningococcal disease (IMD) caused by *Neisseria meningitidis* represents a serious public health concern, with more than 1 million annual cases estimated to occur worldwide [1]. In those with IMD, this normally commensal bacterium (carried asymptomatically in the nasopharynx) is invasive [2]; the precise trigger for this transition is unknown. Patients often present with life-threatening meningitis or septicemia and may progress rapidly from early nonspecific symptoms to unconsciousness and death within 24 h [3, 4]. On average, case fatality rates range from approximately 10–20%, depending on geographic location and meningococcal type, but rates have been observed to be as high as 40% in cases of septicemia; these rates are very high in comparison with other diseases, making any case of IMD an immediate concern [5, 6]. Among those who survive IMD, up to 20% may suffer life-altering sequelae, such as hearing loss, cognitive deficits, skin scarring, and limb amputation [7]. The incidence of IMD is highest in the very young (< 1 year of age), adolescents/young adults, and the elderly [2, 8]. Seasonal fluctuations in IMD rates may occur depending on geographic region, but overall, IMD is variable and unpredictable. Some of this unpredictability may be attributed to the disease incidence remaining low for years at a time until an outbreak is triggered.

Six serogroups (or types) of meningococcus cause the majority of IMD: A, B, C, W, X, and Y; these serogroups are defined based on differing capsular polysaccharides [9, 10]. Vaccination and chemoprophylaxis are the only means of protection against IMD. Several vaccines are currently licensed to protect against disease caused by serogroups A, B, C, W, and Y; a vaccine that includes the serogroup X antigen is in development [11]. Whether a country chooses to implement vaccination with a particular meningococcal vaccine depends on many factors, including local epidemiology, economic considerations, and expert recommendation as well as cost-effectiveness and budget impact. A number of recent cost-effectiveness estimates of vaccines targeting serogroup B IMD have been outside the range of acceptable willingness-to-pay for a quality-adjusted life-year (QALY) threshold using standard methods. However, methods vary among countries (which can lead to differing conclusions among decision-making bodies), and it has been argued that standard approaches may not fully capture the impact of vaccines [12–16].

In March 2018, a panel of experts met in London, United Kingdom, to review methodologies, frameworks and decision-making processes for the evaluation of vaccines, focusing on IMD. Meeting objectives included discussion of vaccine evaluation methods across countries; exploration of IMD prevention benefits that are well-quantified using current methods, not well quantified, or missing in current cost-effectiveness methodologies; and development of recommendations for future vaccine evaluation methods. This report presents an overview of topics discussed, with an emphasis on points of consensus and disagreement among the panel, as well as recommendations made.

## Methods

Panel members included health economists, physicians, patient group representatives, and scientists with extensive experience in the fields of meningococcal disease and vaccine evaluation. Experts were from the United Kingdom, Canada, France, Germany, Italy, the Netherlands, Norway, Spain, and the United States. This manuscript was developed by those attendees who wished to participate as authors.

### Meningococcal vaccines: an international comparison of decision-making processes, frameworks, and methodologies

Methods to evaluate whether a new vaccine offers an efficient use of resources vary from country to country. A rapid evidence assessment of the literature was conducted by 3 authors affiliated with OHE Consulting Ltd (London, UK) to document the evidence used to inform vaccine adoption across countries currently and to specifically determine whether studies of meningococcal vaccine cost-effectiveness have identified and valued various health benefits that could be considered [17]. Based on these results, new criteria and alternative approaches for, and potential improvements to, evaluation methodology were proposed and discussed by the panel.

#### Literature review: rapid evidence assessment

##### Literature search

Three databases were searched for articles published between January 2005 and November 2016 containing keywords related to meningococcal vaccines and economic evaluation: the National Health Service Economic Evaluation Database (UK-based; includes > 16,000 economic evaluations of healthcare interventions), MEDLINE (searched via PubMed), and Google Scholar. Of the 31 articles included in the analysis, most analyzed serogroup B (MenB) vaccines (12/31) followed by quadrivalent conjugate vaccines against serogroups A, C, W, and Y (MenACWY; 9/31). High-income countries such as the United Kingdom (5/31), the United States (5/31), and Canada (3/31) produced the most published evaluations; of the 26 studies that provided full economic evaluations, 7 studies deemed vaccination against MenB, serogroup C (MenC), or MenACWY to be cost-effective; 5 concluded that the evaluated vaccine was not cost-effective; 11 provided mixed results (cost-effectiveness varied based on cost per dose); and 3 were considered unclear (experts were uncertain about criteria for being deemed cost-effective). Across studies, cost per vaccine dose and the influence of herd protection (reduction in disease among nonvaccinated individuals) were the principal determinants of cost-effectiveness.

##### Model type and assumptions

Mathematical model choice varied among the selected articles, with most studies employing simple static models. These models can account only for direct protection of vaccinated individuals, not for herd protection, which is responsible for the majority of cases prevented for some vaccines. Other studies used dynamic models, which are complex but do account for herd protection. Several studies used both model types. Herd protection in cost-effectiveness analyses is an important consideration, especially for adolescent meningococcal vaccination strategies because adolescents are known to have higher carriage prevalence (thus being the principal drivers of transmission) compared with other age groups.

The studies also varied in terms of the assumptions used in their models. Differences in vaccine schedules, age groups targeted for vaccination, and dose numbers highlighted the complexity of cost-effectiveness evaluations that policy-makers must consider when deciding whether to adopt a vaccine. Assumptions also varied regarding how to measure loss of health utility (HU) after contracting a vaccine-preventable disease. Frequently, HU loss is quantified as QALYs in economic studies of IMD. However, consistent estimation of HU loss is difficult to achieve because different instruments used to measure quality of life (QoL) capture different dimensions (e.g., pain, mobility, emotion) and may therefore produce different results. In addition, some HU losses that are the result of late-onset sequelae, such as growth plate damage or more subtle cognitive effects, may not be captured if studies are not conducted over longer time periods. This issue is particularly pronounced for IMD because HU loss among children or adolescents who survived IMD may not be captured fully.

Individual HU losses are also widely variable because sequelae associated with IMD are diverse, can be multiple, and vary in severity. Recognizing these complexities and potential deficiencies, the Joint Committee on Vaccination and Immunisation (JCVI) in the United Kingdom decided that QALYs should be adjusted in situations where health benefits may be overestimated or underestimated by models. The JCVI has applied a QALY adjustment factor (QAF) of 3 (relevant QALY gains are multiplied by 3) [18] to long-term sequelae associated with MenB IMD, which was employed in 2 studies identified in the literature search. Studies of HU loss typically address only the patient and his or her sequelae, but more recent assessments have included QALY losses for caregivers to reflect the wider impact of IMD on the network of individuals surrounding the patient [13, 19].

Discounting can be considered the inverse of the interest rate applied to future money and health [20] and is meant to account for the time horizon used in mathematical models for vaccine cost-effectiveness. Although use of discount rates is standard practice in health economic analyses, discounting methodology has been criticized in vaccine modeling relative to other healthcare interventions because of the timing of costs and benefits [21, 22]. Vaccine program costs occur early in the model, while the benefits (i.e., reductions in disease[s] the vaccine is meant to prevent) may not be evident for several years; thus, the benefits of vaccination are more heavily discounted compared with the costs, making vaccination appear less favorable compared with health programs where benefits are realized quickly.

Discount rates may be applied equally or differentially to costs and benefits. Applying a lower discount rate to benefits compared with costs may be done to account for the growing value of health effects [23]. Application of different discounting rates can lead to wide variation in the incremental cost-effectiveness ratio (ICER) provided by a model [24]. For example, when equal discount rates of 4% were applied to an analysis of human papilloma virus (HPV) vaccination in the Netherlands, the ICER was estimated at €101,700; however, applying discount rates of 4% and 1.5% to costs and health effects, respectively, the ICER decreased to €29,900 [24]. Regarding IMD vaccination specifically, 1 study found that application of a 5% discount rate compared with a 0% discount rate led to an ICER increase of 419% for a quadrivalent meningococcal conjugate vaccine [25]. Discounting as a principle is widely accepted. However, application of differential discounting varies by country and is the subject of ongoing debate [26]. Nevertheless, more countries are opting to use differential discounting in their cost-effectiveness analyses [21].

The use of differential discounting in models also varies by country. Although more countries are moving toward differential discounting, most still use equal discount rates. Germany uses both types of discounting and compares results between them, whereas the United Kingdom applied a 3.5% discount rate equally to costs and benefits (although this policy is being reconsidered regarding vaccines and has since been reduced to 1.5% for all health costs and benefits) [27–29]. Panel members concluded that there is no straightforward answer to the question of whether differential discounting better reflects the value of vaccines relative to equal discounting. If used, differential rates should be tested in sensitivity analyses to inform decision-makers about the relative importance of selected discount rates to the outcome. Panel members also suggested that additional discussion is needed to address whether vaccines specifically should merit application of differential discounting rates or whether all health technologies with a lengthy benefit time horizon should benefit from differential discounting.

Incremental analyses are used to estimate additional costs and benefits beyond a specified intervention and may be beneficial when evaluating differences in vaccine dose schedules or targeted age groups. Incremental analyses and use of lifetime timescales remain well-accepted strategies for economic evaluation of vaccines. For instance, in an evaluation of MenC vaccination in the Netherlands, incrementally comparing a 3-dose series in infancy to a single toddler dose at 14 months of age, approximately 2 extra deaths were predicted to be averted by implementing the 3-dose schedule, with the single-dose schedule averting the vast majority of 20 expected deaths. This suggested that implementation of an infant series would not be cost-effective, and a single-dose schedule after the first birthday was chosen [30]. In the United Kingdom, a separate internal analysis indicated that all schedules were cost-effective, and that country chose to use a 3-dose infant MenC schedule with a catch-up campaign to 21 years. Incremental analyses may also be used to assess whether adjustments to existing schedules will be cost-effective, although the choice of comparator is complex in the context of highly effective programs where little ongoing disease remains.

Most studies identified in the literature search (61%) attempted to conduct analyses from the societal perspective, which seeks to account for all costs and benefits irrespective of whom they are associated with (however, most identified analyses were not able to capture all indirect benefits comprehensively). This perspective encompasses the indirect costs of IMD that include economic productivity losses, premature death, inability to work, and special educational or social welfare needs for children with cognitive or sensory sequelae and their families. Such indirect costs are important to consider when evaluating a preventive health intervention, such as vaccination.

Similar to observations regarding models and discounting, the preferred analysis perspective for cost-effectiveness evaluations of vaccines varies from country to country. Although selection of perspective is related to the goal of the evaluation (e.g., a health ministry aiming to optimize public health impact from a fixed budget allocation), experts suggested that conducting the most comprehensive analysis possible using the widest perspective should benefit both decision-making bodies and the public.

Current models for vaccine evaluation include static and dynamic models, with the latter capturing possible herd effects of vaccination programs. Different countries prefer different models, but experts suggested both model types could be used and results compared to provide a more comprehensive view of vaccine cost-effectiveness. Dynamic models must be used to appropriately capture all benefits for vaccines that prevent transmission as well as disease. Regarding meningococcal vaccination, additional studies will be required to discern whether herd effects play a role in the effectiveness of MenB vaccination programs, as has been demonstrated for vaccines targeting other serogroups [31–34]. A cluster-randomized study is currently under way in Australia to investigate MenB-4C (Bexsero^®^, 4CMenB; GlaxoSmithKline Vaccines, Srl, Siena, Italy) for effects on carriage, and by extension, transmission [35]. Results from this study coupled with an adolescent study under way in the United Kingdom [36] may elucidate whether adolescent MenB vaccination programs could produce a herd effect.

The choice of analytic time horizon used in models is also subject to debate; panel members suggested that a cohort lifetime horizon seems to be preferred in vaccine cost-effectiveness analyses, but payers may apply a shorter horizon. If a shorter horizon is included, such as with a cross-sectional approach useful for evaluating budget impact and health at the population level, relevant outcomes may not be captured. However, extending the horizon long into the future may lead to increasing uncertainty surrounding estimates [37].

#### Country comparisons: methodology for health technology appraisal and decision-making

Evidence and procedures used to evaluate health technologies vary by country. The OHE consulting report documented methodology and criteria used by 7 countries (Australia, France, Germany, Italy, Japan, New Zealand, and the Netherlands; Table 1) to inform policy decisions surrounding vaccine adoption, which included interviews with country-specific experts. Experts were those individuals with a deep knowledge of the processes followed for vaccine adoption in that country and/or direct understanding of formulary development or vaccine-related reimbursement decisions. Countries were selected if they had conducted an economic evaluation of a MenB vaccine or if their evidence base and decision-making processes were advancing rapidly (the United Kingdom was not included in the analysis based on the original scope of the project).

**Table 1:** Methodology of vaccine health technology appraisal

Country	Health technology appraisal method
Australia	Decisions to approve vaccine reimbursement rest with the Department of Health (DoH) within the federal government. The Pharmaceutical Benefits Advisory Committee (PBAC) provides guidance to the DoH regarding whether to include the vaccine in the Pharmaceutical Benefits Scheme or the National Immunisation Program. The Economic Sub-Committee of the PBAC is responsible for evaluating and interpreting economic analyses of health technologies; this guidance, combined with additional input from the Australian Technical Advisory Group on Immunisation and another independent group (frequently academic health economists) is used for comprehensive assessments of vaccine applications. Uncertainty surrounding economic evaluation outputs is acknowledged and clearly considered in final decision-making
France	Vaccination policy in France is developed by the Ministry of Health based on recommendations from the Technical Vaccination Committee (CTV), an independent expert committee that devises immunization strategies and provides advice—including economic evaluations—on new vaccines. The CTV provides pharmacoepidemiologic evidence, disease modeling, and assessment of vaccination strategies during dossier evaluation. If deemed necessary by the CTV, vaccine cost, program cost, affordability, or financial sustainability may also be evaluated. After consideration of evidence from the CTV and the Commission for Transparency, which determines the impact of the vaccine on public health services, the final decision regarding integration into the national immunization schedule is made by the High Authority of Health
Germany	In Germany, vaccination recommendations at a national level are made by the Standing Committee on Vaccination (STIKO). Based on the STIKO recommendations, the Federal Joint Committee (G-BA) decides whether a vaccine is included in the mandatory service of statutory health insurances. STIKO recommendations principally rest on a risk–benefit assessment that includes a number of criteria described in a standard operating procedure. The criteria list comprises, among others, efficacy, effectiveness, safety, number needed to vaccinate, and expected population-level effects. Economic analyses are not obligatory but may be considered when based on independently funded models. QALYs can be used as a summary measure of health outcome, although the STIKO and the G-BA have not set a cost-per-QALY threshold
Italy	The recommendation of health technologies in Italy is conducted at the national level, but because of a highly decentralized national health service, decisions by the National Ministry of Health are nonbinding on local health authorities. The National Vaccine Prevention Plan issued by the Ministry of Health provides guidance and recommendations at the national level. However, decisions surrounding vaccine adoption may differ from region to region because some vaccines are mandatory at a national level and administration of others is determined within each region. Italian guidelines use cost-effectiveness analyses and QALYs as the foundation of economic evaluations
Japan	Regulation of vaccines and other heath technologies requires coordination between the Japanese Ministry of Health, Labor, and Welfare (MHLW) and the Pharmaceuticals and Medical Devices Agency. In 2009, the Infectious Disease Sectional Committee made a recommendation to the government leading to inclusion of economic evaluations in decisions regarding vaccine adoption. The Health Science Council, also within the MHLW, is responsible for considering cost-effectiveness, but this evaluation is not required
New Zealand	The Pharmaceutical Management Agency (PHARMAC) in New Zealand decides the public funding of pharmaceutical products, including vaccines. PHARMAC evaluates 4 parameters when assessing a product: need, health benefits, costs and savings, and suitability. In addition, the agency considers 3 levels of impact on society: personal, health sector, and wider society. Information also considered for vaccine efficacy modeling are degree and length of protection, age at administration, dosing schedule adherence, adverse reactions, possible loss of potency due to cold chain issues, and herd protection. The final decision for vaccine adoption rests with the Minister of Health based on the PHARMAC evaluation
The Netherlands	The Dutch Ministry of Health decides whether to add a new vaccine to the national immunization plan and/or the “positive list” based on guidance from a Health Council subcommittee and the Drug committee, respectively. The Health Council subcommittee considers 2 assessments, the first by the National Institution for Public Health and Environment (RIVM), and the second, often by a university. One main criterion for evaluation are economic analyses. The Drug committee conducts 1 assessment, using materials submitted by the vaccine manufacturer, which must include a cost-effectiveness analysis. Recently, a new policy has been implemented to streamline the evaluation process to include only the RIVM and manufacturer-sponsored assessments. Vaccine evaluation is prioritized by the Ministry of Health, based mostly on severity and health burden of the disease in question, whereas evaluation by the Drug committee for inclusion in the positive list is not prioritized but initiated by the manufacturer. Recommendations of the Health Council subcommittee and Drug committee include information about the degree of uncertainty in the analyses and how this uncertainty may influence the robustness of the outcomes; a vaccine is not introduced if uncertainties are too influential or the severity is not considered high enough

*CTV* Technical Vaccination Committee, *DoH* Department of Health, *G*-*BA* Federal Joint Committee, *MHLW* Ministry of Health, Labor, and Welfare, *PBAC* Pharmaceutical Benefits Advisory Committee, *PHARMAC* Pharmaceutical Management Agency, *QALY* quality-adjusted life-year, *RIVM* National Institution for Public Health and Environment, *STIKO* Standing Committee on Vaccination

##### Expert interview results

Across all 7 countries, clinical outcomes and the targeted age group are formally required to be considered in decision-making. All countries have evaluated HU loss measured by QALYs, although in Germany this is part of an economic evaluation that may not always be required; in Japan, this type of evaluation is uncommon and informal (i.e., the evaluation is not normally part of the analysis but has been considered in particular cases). Likewise, the assessments of cost-effectiveness and disease burden, severity, and sequelae are formally considered in all countries except Japan, although they remain key criteria in Japan’s decision-making. France and Japan do not require consideration of the cost offset per patient to the healthcare system, but Japan does commonly and informally consider this parameter. Consideration of public preferences and innovation aiding vaccine development, although not required, is commonly considered in Japan but not in any of the other countries. The concept of equity, which encapsulates efforts to reduce inequality in access to healthcare, was considered a key component of decisions in Australia, Japan, and New Zealand, but is not formally considered. Consideration of “peace of mind” benefits was uncommon.

Meningococcal vaccine recommendations differ by country, with the MenC vaccine included in the national immunization schedule in Australia, France, Germany, and Italy, but not New Zealand or Japan. MenC vaccine was recommended in the Netherlands until the spring of 2018, when MenACWY was recommended instead [38]. The MenACWY vaccine is also included in the national immunization schedule in Italy but not in Japan, Australia, France, Germany, or New Zealand. Among the 7 countries reviewed, MenB vaccination is routinely recommended only in Italy for children ≤ 1 year of age.

Adding to the country-specific variability in vaccine evaluations and approvals, individual countries will negotiate vaccine prices with manufacturers based on the laws and guidelines specific to that country. If a manufacturer has additional drugs in its portfolio in a given country, negotiations may include adjustment of other drug prices to provide more financial leeway for the vaccine price negotiation. Some countries, such as New Zealand, operate on fixed budgets for pharmaceuticals, including vaccines, whereas others do not.

### Measuring disease burden

Based on the OHE consulting report, disease burden is a crucial factor considered by 6 of the 7 countries when evaluating a vaccine for inclusion in a national immunization program. Burden of disease and its sequelae can be clinical (incidence, morbidity, mortality), economic (costs of the disease and sequelae), or humanistic (QoL). Determination of true incidence relies on the accurate capture of case numbers, but trends in incidence may also make use of sentinel cases (cases from selected reporting sites). For the purposes of economic evaluations, health-related burdens (morbidity and mortality) are commonly quantified as QALYs or disability-adjusted life-years. Broader and narrower aspects of burden included in the QALY will influence the estimate. For meningococcal disease, burden may differ depending on whether a model incorporates acute and/or long-term QALYs and QALYs from the survivor’s and the caregiver’s perspective. The OHE consulting report recommends rigorous assessment of the data used to generate QALY loss estimates and determination of whether the estimates can be transferred across different disease contexts (e.g., different countries).

Panel members discussed possible factors to consider in vaccine evaluations to achieve a comprehensive view of the burden of meningococcal disease. In the Netherlands, mortality is the main factor considered in burden estimates for vaccine evaluations, whereas Germany focuses on hospitalization, long-term disability, and mortality. France considers the severity of the disease itself to be most important, whereas QoL is viewed as a less important factor. Italian authorities place more focus on morbidity than mortality, viewing costs related to social burden and use of national health service resources (including possible need for life-long services) as main contributors to decision-making. The panel suggested that subsequent comorbidities triggered by IMD is an overlooked component of disease burden, given the potentially lifelong multiple sequelae experienced by survivors.

A critical factor in determining whether to adopt a vaccine lies with the accurate quantification of disease burden, which directly influences the predicted impact of the vaccine program. For some vaccine-preventable diseases, such as HPV and rotavirus, the disease burden is relatively predictable.

In contrast, IMD is highly unpredictable over time and panel members agreed that a key challenge is how to incorporate that unpredictability into a mathematical model. Using MenC vaccination in the United Kingdom as an example, disease incidence decreased after vaccine implementation as expected, but the panel agreed that predicting the true annual incidence in the absence of the vaccination program would have been impossible. The consensus framework for disease modeling is to assume a steady-state annual incidence in the absence of vaccine intervention. Thus, vaccination benefit is measured as the predicted reduction in cases. In the case of IMD, disease incidence can remain low for many years until the emergence and expansion of hyperendemic virulent clones. Models are unable to predict when this may occur. Outcomes also depend on the selected time horizon; years of low incidence can dominate modeling outputs even if hyperendemic periods may be a public health concern; thus, the stochastic nature of IMD calls into question whether the same models should be used for predictable and unpredictable vaccine-preventable diseases. Experts agreed that vaccines for diseases with epidemic potential should be evaluated differently depending on disease predictability, although the panel could not identify an existing methodology to address this.

Experts also agreed that IMD outbreaks vary in severity depending on serogroup or clonal complex, which may translate to more severe sequelae that may not be captured and quantified in analyses depending on the data sources used. The panel agreed that evaluation of aggregate IMD, rather than evaluation of burden associated with a specific meningococcal serogroup, is suitable for cost-effectiveness models, similar to analyses performed for aggregate pneumococcal disease rather than analysis by specific pneumococcal serotype. Unlike pneumococcal disease, for which herd effects are now well known, if data are not available to inform whether a vaccination program produces herd protection, the unpredictability of transmission effects adds to the uncertainty of the vaccine impact. These issues continue to present challenges in vaccine cost-effectiveness studies, and experts indicated a need to identify strategies to incorporate these uncertainties into evaluations.

Capturing burden of disease requires more than simply capturing case numbers. The burden of IMD is likely to be underestimated in economic analyses, because QALYs may not reflect all types of burden (e.g., burden for the patient, the caregiver, individuals with multiple sequelae rather than a single sequela, pediatric versus adult populations). The QoL profiles used to generate QALYs are measured using patient reported outcomes such as the EQ-5D and other tools such as time trade off, the visual analogue scale and the standard gamble method [39]. These instruments measure and apply values to attributes of health, including anxiety. However, the panel did express concern regarding the ability of these instruments to capture infant health status based on proxy respondents, such as parents. The panel also questioned the evidence base estimating QALY loss later in life and whether such evidence is adequately included in cost-effectiveness analyses.

When considering input parameters that should be included in cost-effectiveness models, experts suggested that health outcomes (e.g., sequelae, mortality), accurate assessments of infant QoL, and impact on caregivers (during acute disease and later in life) should be incorporated. Attempting to measure all possible effects of IMD on QoL is difficult because impacts on caregivers decrease as social distance from the IMD survivor increases [40]. However, focusing on the IMD survivor and his or her immediate family would likely be sufficient to account for QoL-based QALY loss associated with an IMD case. In addition to QALYs, elements such as peace of mind (decreased anxiety) may merit inclusion in models, although evidence of this in relation to vaccine-preventable diseases is currently limited.

### Consideration of vaccine benefits

Discussions to develop a list of vaccine benefits that should be included in current meningococcal vaccine evaluations centered on analysis perspective, inclusion of indirect benefits, and the ease with which various benefits may be measured.

Before addressing benefits, the panel first discussed inclusion of indirect costs in meningococcal vaccine evaluations. These factors are important but typically not considered because of the difficulty associated with measuring them. Examples of indirect costs include lost productivity of the survivor (if of working age) or caregiver, and lower educational attainment by younger survivors. Lost productivity may be larger for the IMD survivor in the workforce as opposed to a retired individual, but it is important to note that the elderly population still engages in economic activity (e.g., volunteering, informal care) that is otherwise lost during illness with IMD. Lost productivity may be a crude proxy for societal costs, especially for a disease such as IMD that predominantly affects children. Future earnings potential for children once they reached working age would be dramatically reduced (depending on the discount rate used), potentially leading to equality issues if only productivity losses were used as a proxy for societal costs.

Although many countries do not currently consider indirect costs, experts agreed that indirect costs should be included in evaluations, not only for those vaccines that target IMD, but also for vaccines in general. Indirect costs may be considered in the form of a societal cost-effectiveness analysis. Multicriteria decision analysis (MCDA) is an alternative method of health technology assessment that can be applied to vaccines. This method involves defining the criteria that are important to recommendation or reimbursement decisions, scoring the vaccine on each criterion, assigning a weight to each criterion specified, and constructing an overall vaccine score. Decisions are made by comparing the score to some prespecified threshold or to the scores for other candidate vaccine or nonvaccine interventions. Although MCDA is evolving to become more rigorous, it is often criticized for the ad hoc manner in which preference weights are assigned and for the unintentional double-counting of overlapping criteria.

One major vaccine benefit is herd protection. In the example of MenC vaccination in the United Kingdom, herd protection was not initially predicted as a benefit following implementation of the vaccine, but it was later shown to have played an important role in the overall effectiveness of the MenC program. Herd effects are relatively straightforward to measure after vaccine implementation but are difficult to predict and therefore to model accurately beforehand. The size of herd effects from MenB vaccination in adolescents is not yet clear, but additional studies designed to address this issue are currently under way.

Numerous indirect benefits may be considered in cost-effectiveness analyses of meningococcal vaccines conducted from a societal perspective (Table 2). Among these, labor force benefits and educational benefits are more easily recognizable, but less well-known benefits, such as increased equity and decreased nosocomial infections, are also evident. Cost-effectiveness analysis allows comparison of interventions (with different success indicators) within and between sectors. However, this advantage may be mitigated by potential ethical issues stemming from the assignment of greater value to reducing the mortality or morbidity risks of high-earners compared with low-earners (all else being equal) [41].

**Table 2:** Indirect benefits of meningococcal vaccination programs in a cost-effectiveness analysis from a societal perspective^a^

Benefit	Description
*Economic and financial*	
Labor force	Increased productivity and tax revenues
Education	Increased school attendance leading to better education and greater potential productivity as an adult
Medical care	Lower use of healthcare resources
Outbreak control	Lower expenditure on outbreak response
Indirect costs	Lower mortality associated with meningococcal disease (cause of death shifts to other diseases with different associated costs)
*Health*	
Herd effects	Reduction in disease incidence among unvaccinated individuals due to decreased transmission of the pathogen
Comorbidities	Lower risk of developing secondary comorbidity associated with primary disease
Nosocomial infections	Lower risk of acquiring additional infection while hospitalized for primary disease
Antimicrobial resistance	Reduced incidence of resistant infections in the population due to reduced reliance on antibiotics
Health systems	Less strain on health systems, increasing capacity to treat other conditions
*Societal*	
Family members/caregivers	Lower burden (financial, psychological) on those caring for survivors
Leisure	Improved quality of leisure time due to better health
Peace of mind	Less anxiety at the individual and societal level about contracting meningococcal disease
Equity	Increased socioeconomic equity and increased access to preventive care for disadvantaged groups
Political implications	Lower infant/child mortality is correlated with stable political systems

^a^Indirect benefits include non-health benefits that follow from reduced incidence of meningitis; they also include downstream health benefits

The ability to quantify and monetize indirect benefits of vaccination plays a substantial role in producing accurate evaluations of vaccines for adoption. Panel members agreed that indirect benefits should always be considered in vaccine evaluations, but uncertainty still remains surrounding the availability of information needed to fully consider these types of benefits. Including the wider societal benefits of preventing ill health could ultimately lead to higher willingness-to-pay for health interventions in general.

### Vaccine benefits not commonly considered

**Fig. 1:**
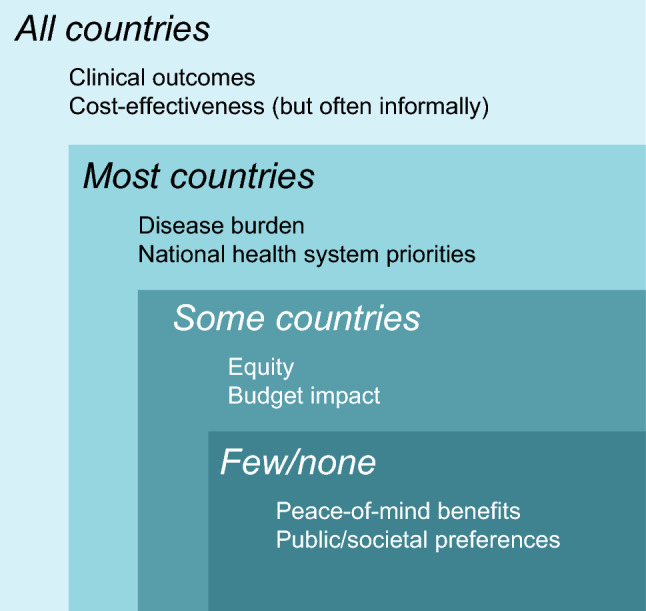
Decision criteria used in meningococcal vaccine evaluations. Criteria used in vaccine evaluations vary by country. Examples are shown for those that are more or less frequently considered

In addition to the clinical and economic benefits offered by meningococcal vaccines, less frequently considered benefits may be important factors in determining whether to add a vaccine to a national immunization schedule (Fig. 1). For example, vaccination may provide peace of mind (decreased anxiety) to patients (and caregivers) who benefit from knowing they have reduced their risk of contracting a potentially fatal disease. Peace of mind is difficult to measure because, among those who experience it, peace of mind accrues to healthy individuals rather than to those with the disease, and is not usually included in base/reference cases in standard economic evaluations of vaccines. It is not clear whether or how decision-makers should take into account healthy individuals’ concerns regarding the risk of severe illness for themselves or their family members. Stated preference research may aid health economists in understanding how people assign value to this type of intangible benefit.

In a qualitative study conducted by 2 panel members and their colleagues, 21 UK adults participated in 4 rounds of semistructured interviews to identify factors they considered important to vaccine decision-making [42]. Five factors emerged as dominant: age group, disease severity, how common the disease is, effect on caregivers, and social group (advantaged/disadvantaged). Vaccine side effects, herd effects, decreased disease incidence, and peace of mind were not considered as important. A subsequent focus group indicated that vaccination may be viewed by some as more a matter of accepted routine than for seeking peace of mind. Although these findings are informative, additional studies are needed to confirm these observations before clear conclusions may be reached.

Related to peace of mind is utility in anticipation, which describes the provision of the peace of mind benefit to vaccinated individuals and their caregivers starting at the moment of vaccination, when they know they have protected themselves from disease [43]. Earlier vaccination increases the duration of the utility of anticipation benefit. However, standard economic approaches do not include this parameter but rather count the benefits from the moment of illness occurring. Arguably, current economic evaluation methodology omits the additional benefit of anticipation that occurs before illness and therefore underestimates total vaccine benefits. Moreover, including peace of mind benefits only for the proportion of the population predicted to develop the disease potentially underestimates peace of mind for all who are vaccinated against something they fear, whether or not those individuals would have developed the disease. A more comprehensive value of vaccine benefits should include prevented health loss from time of illness onset plus the utility in anticipation benefit that initiates at vaccination.

#### Weighting of quality-adjusted life-years

QALY weights based on societal preferences are not typically applied in vaccine evaluations. Many decision-making bodies consider all QALYs to be of equal social value, but there is some evidence to suggest that society would prefer to weight QALYs differentially based on severity of illness and the age of the affected population (i.e., there is a greater willingness to pay for interventions that prevent QALY loss in severe disease rather than in mild disease and in infants rather than adults) [44–49]. Conversely, ethicists may not agree with such societal preferences; for example, in the United Kingdom, a policy statement for responding to pandemic influenza states that everyone matters equally (i.e., equal importance is placed on the lives of the elderly as the lives of children) [50].

Although societal preferences indicate disease severity should be considered in vaccine evaluations, the additive nature of a QALY (i.e., preventing small losses in QoL in many people can be worth as many QALYs as preventing life-long disability in few people) may require that severity be prioritized through a separate weighting. In addition, studies conducted to assess public preferences for preventive versus curative interventions indicate greater value is placed on preventive treatments, especially for more severe and possibly life-threatening illnesses [51–53]. These studies provide support for the possible public perception of meningococcal vaccines as highly valuable because they meet the criteria outlined above: meningococcal vaccines are preventive, protect against a severe, life-threatening disease, and offer protection to targeted vulnerable populations.

Some members of the group agreed that QALY weighting could be a useful way to reflect the preference of society in vaccine evaluations, although the influence of weighted QALYs on decision-making is often unclear to those not participating in decision-making processes, suggesting a need for increased transparency. For example, the JCVI assigned an adjustment factor of 3 to QALYs determined for long-term sequelae due to IMD, though the rationale for this was not published at the time. As an alternative to QALY weighting, decision-makers could explore alteration of the cost-effectiveness thresholds [54] for vaccine adoption, giving particular consideration to meningococcal vaccines because of the severity and consequences of IMD in pediatric populations.

#### Peace of mind benefits

Quantifying peace of mind is complex, and uncertainty persists regarding how and when to include such a parameter in vaccine evaluations. The panel debated whether peace of mind is essential to cost-effectiveness analyses or whether its influence is insufficient to sway outcomes. To benefit from peace of mind, a patient or caregiver must first be aware that a threat exists. Some parents may be unfamiliar with the incidence rates and clinical consequences of meningitis and some may have an exaggerated perception of the likelihood of contracting IMD. Conversely, meningococcal vaccination may potentially allay a parent’s fear of meningitis each time his or her child develops a fever or nonspecific symptoms that overlap with early-stage IMD, but this remains a matter of debate. Whether vaccination could prevent inappropriate use of healthcare resources due to peace of mind benefits is unclear, but the costs saved by preventing diversion of healthcare resources could potentially be quantifiable, although they are not factored into cost-effectiveness analyses.

Studies using revealed preferences and evaluation of willingness-to-pay for meningococcal vaccines are possible strategies to understand how much importance society places on preventing IMD. However, using propensity to pay as a methodology would need to account for equity issues related to wealth and perception of payment for health, rather than viewing the stated propensity in isolation. Panelists also discussed surveying parents who sought medical care for their child for suspected meningitis as a possible strategy to obtain estimates of unnecessary resource use. Overall, how to quantify and include peace of mind is worth additional exploration, but panelists did not agree on its usefulness.

### Are current tools sufficient, or do we need to use adjustment factors?

As noted above, to account for underestimation of true health benefits in models of MenB cost-effectiveness, the JCVI introduced a QAF of 3 for long-term or lifelong sequelae (the QAF was not applied to short-term losses). Factors influencing the QAF included difficulty capturing loss of QoL in IMD survivors, especially the youngest individuals, the innovative nature of the MenB vaccine being evaluated, and wider societal preferences. When comparing modeling outputs using a QAF of 3 versus no QAF for a 2-, 3-, 4-, and 12-month MenB vaccination schedule, the number of QALYs gained increased substantially whereas cost per QALY gained and vaccine price for cost per QALY gained (threshold set at £20,000) decreased. Essentially, application of the QAF rendered the vaccine cost-effective at £3 per dose [12].

Panel members discussed whether the current tools for measuring disease burden and vaccine benefits are sufficient to provide accurate analyses and whether adjustment factors should be used more widely in the assessment of vaccines. However, there was no clear consensus on the use of the QAF.

Some panelists viewed the QAF as an arbitrary, nontransparent adjustment that should not supplant addressing the underlying issue of the imperfect nature of QALYs used in cost-effectiveness models. The assigned value of the QAF (× 3) is also a subject of debate. Some of the panelists suggested that cost-effectiveness thresholds could be increased as an alternative to application of the QAF. In addition, consideration of the QAF encompassing the value of vaccine innovation was viewed by some to be a parameter that should instead be factored into the cost-effectiveness threshold. Although increased thresholds could potentially be applied for only certain types of interventions with specific characteristics (such as vaccines), they may cause special treatment to be given to new interventions (compared with displaced existing interventions with similar characteristics) as an unintended consequence. Additionally, flexibility to make adjustments to the threshold may result in multiple thresholds being used to evaluate cost-effectiveness of different health interventions.

Other panel members suggested the QAF could be used effectively because it is not applied universally but is considered only for specific situations, such as IMD vaccines; the sense of urgency associated with efforts to prevent IMD could justify use of the QAF. Moreover, the JCVI had already noted that application of the QAF was equivalent to raising the cost-effectiveness threshold to £45,000 [18]. Nevertheless, a clear process should be implemented to determine in which situations the QAF should be applied.

Factors that may be difficult to capture using current cost-effectiveness analysis tools include equity, political factors, innovation, and other generally intangible parameters. This discussion highlighted the remaining uncertainty surrounding how to optimize the assessment of vaccine cost-effectiveness. An additional review of tools currently used to measure disease burden is needed.

### Consistency and transparency in vaccine decision-making

During evaluation of a vaccine for adoption, numerous variables are considered that range in transparency and consistency. Those variables that are more consistent or predictable are frequently more transparent and are often included in formal considerations of vaccines. Such variables are more easily quantifiable and include clinical outcomes, severity of disease, burden of disease, and associated sequelae. Factors that are less consistent tend to be less transparent and more difficult to quantify. These include equity and public preference, which are encompassed in the public’s understanding of the impact of disease on their own lives.

As demonstrated in the OHE consulting report on vaccine decision-making processes in 7 countries, substantial variability exists in evidence considered and methods used to inform vaccine adoption. Panel members discussed advantages and disadvantages associated with increasing the consistency and transparency of vaccine decision-making.

Economic evaluation models are generalizable and the results should be transferrable across countries with similar meningococcal epidemiology; panelists agreed that a core set of principles for a single model could be developed. However, there are issues particular to each country that complicate universal application, such as variable disease burdens, changing epidemiology, differences in disease surveillance systems, disparate healthcare laws, differential budget constraints, societal preferences, and political factors, all of which influence consistency in evaluating vaccines for adoption. As outlined by the World Health Organization guidance on the standardization of economic evaluations for vaccine programs [55], such a model could be customizable per country-specific inputs, leading to a consistent evaluation process that produces appropriately variable (for each country) outcomes.

Experts agreed that transparency in decision-making is beneficial to the public and should be implemented wherever possible. The panel suggested that making mathematical models available for critique may both increase transparency and improve any given model [56]. Inclusion of stakeholder input into the evaluation process may also enhance transparency. An example is the UK JCVI, which includes 2 lay representatives who are interviewed and chosen to bring nontechnical insight into vaccine policy and consequent public health impact. However, experts noted that involvement of patient groups in vaccine decision-making may be complicated, but patient groups that have expert knowledge can bridge the gap between patients/families and experts, and could play a role in advising decision-makers. Additional assessments of the value of patient/public involvement are needed.

## Conclusions

Panel experts in health economics and meningococcal disease met in 2018 to review current information on meningococcal vaccine cost-effectiveness evaluation strategies. Fundamentally, the unpredictability of IMD hinders a precise evaluation of meningococcal vaccines for inclusion in national immunization programs; however, transparent, dynamic, broad, and flexible health-economic models do provide the necessary evaluative tools for decision-making bodies such as JCVI to evaluate cost-effectiveness of vaccines. This can in turn inform technical committees regarding the value of adding new vaccines into routine schedules. Priorities for future discussion are enhancing consistency of frameworks for evaluating outcomes of vaccine introduction; reviewing existing tools used to capture QoL; developing a modeling framework to address the unpredictability of IMD; determining how indirect costs and benefits (i.e., carer health benefits) are considered within models; and assessing how the weighting of QALYs and cost-effectiveness thresholds may affect the use of adjustment factors such as the QAF.
